# Workplace Environment and Psychological Distress of Nurses and Nursing Assistants During COVID-19

**DOI:** 10.1177/10547738261429339

**Published:** 2026-03-23

**Authors:** Harry Adynski, Cassandra Dictus, Mary K. Killela, Gillian I. Adynski, Elizabeth Allen Myer, Leah Morgan, Hayden Hmiel, Jessica R. Williams

**Affiliations:** 1 University of California, San Francisco, USA; 2 Duke University and the Durham VA, NC, USA; 3 University of Utah College of Nursing, Salt Lake City, USA; 4 Duke University School of Nursing, Durham, NC, USA; 5 Research Triangle Institute (RTI) Health Solutions, Research Triangle Park, NC, USA; 6 Queens University of Charlotte, NC, USA; 7 University of North Carolina at Greensboro, USA; 8 University of North Carolina at Chapel Hill, USA

**Keywords:** perceived work environment, occupational stress, mental health, psychological distress, nursing staff

## Abstract

Registered nurses (RNs) and nursing assistants (NAs) play critical roles during crises. Understanding how the perceived work environment affects psychological distress among these healthcare workers is essential for developing interventions that support mental health and organizational outcomes. This study examined the association between perceived workplace environment and psychological distress, including stress, depressive, anxiety, and somatic symptoms, among inpatient RNs and NAs during the COVID-19 pandemic. A cross-sectional survey was conducted with 84 RNs and NAs employed at a large academic medical center between April and September 2021. The Practice Environment Scale of the Nursing Work Index (PES-NWI) was used to measure the perceived workplace environment, while psychological distress symptoms were assessed using the Perceived Stress Scale, Patient Health Questionnaire-9 (PHQ-9), Generalized Anxiety Disorder-7, and PHQ-15. Descriptive statistics and independent t-tests analyzed cohort characteristics. A stepwise approach with univariate and adjusted linear regression models assessed the relationship between workplace environment scores and psychological distress, incorporating demographic variables. Approximately one-third of participants reported moderate to severe psychological distress. No significant differences in distress symptoms were found between RNs and NAs; however, NAs rated Collegial Nurse–Physician Relations significantly lower than RNs (*t* = −3.84, 95% confidence interval [*CI*] [−1.15, −0.33], *p* = .001). Across all unadjusted models, higher PES-NWI total scores were associated with lower psychological distress. In adjusted models, unit-level Nurse Manager Leadership and Support subscale was significantly related to lower depressive (estimate: −2.03, 95% *CI* [−4.01, −0.04], *p* = .045) and somatic symptoms (estimate: −2.11, 95% *CI* [−4.16, −0.06], *p* = .044). These findings underscore the critical role of nurse managers in fostering supportive workplace environments and highlight the unique challenges faced by NAs. Targeted interventions at the unit and hospital levels can enhance RN and NA well-being, improve organizational outcomes, and build a resilient nursing workforce equipped to navigate future crises.

## Introduction

During times of crisis, registered nurses (RNs) and nursing assistants (NAs) are on the front lines, providing crucial care and support to those affected. The COVID-19 pandemic significantly impacted health systems and tested the capacity of the nursing workforce to respond. RNs and NAs experience increased stress during these emergencies, which can have lasting effects on patient safety, patient satisfaction, quality of care, and the healthcare system as a whole (Jun et al., 2021; L. Z. Li et al., 2024; Shiao et al., 2007). Inpatient RNs and NAs played a key role in providing care to COVID-19 patients during case surges. Simultaneously, other departments saw low censuses while patients avoided healthcare settings (Mattingly et al., 2021). These rapid changes likely increased work-related stress for RNs and NAs, regardless of whether they were directly caring for COVID-19 positive patients (Kim & Yang, 2023). Research has shown that such stress can lead to burnout, compassion fatigue, and moral distress among nurses (Jarden et al., 2020). Furthermore, low resilience, a key factor in coping with workplace stress, has been linked to negative organizational outcomes such as increased nurse turnover (Nantsupawat et al., 2017). This turnover is particularly costly for healthcare organizations, both financially and operationally (Y. Li & Jones, 2013).

Several studies have reported on nurses’ stress and coping in past crises. During Taiwan’s 2003 SARS outbreak, nurses felt increased stress, feared exposure at work, and many sought jobs outside healthcare (Shiao et al., 2007). More recently, there have been reports of high anxiety, depression, and insomnia in nurses caring for patients with COVID-19 (Bostan et al., 2020; Lai et al., 2020). In a descriptive study of 230 medical staff in China during COVID-19, nurses were more likely than doctors to experience anxiety and female health workers were more likely than males to experience post-traumatic stress disorder symptoms (Huang et al., 2020). While not the sole factor affecting nurses’ anxiety, the work environment can play a crucial role in mitigating anxiety symptoms. A prior study indicated that RNs perceptions of their work environment significantly impacted their intention to leave (Adams et al., 2021). Therefore, a greater understanding of nurses’ perceptions of their work environment can identify areas for intervention and ultimately improve workforce well-being and retention. Nursing staff psychological distress is a critical clinical outcome, as untreated distress not only increases the risk of healthcare workers needing mental health care themselves, but also can further strain healthcare system outcomes such as patient care quality, safety, and satisfaction, along with staff retention, productivity, and organizational performance.

To date, the literature has not adequately explored the role of perceived workplace environments in the stress experienced by RNs and NAs during the pandemic. Furthermore, the literature has largely focused on the RN nursing workforce, excluding the NA workforce, which highlights a significant gap. Due to the responsibilities of NAs (e.g., patient personal care, assistance with meals), their exposure and infection risk during COVID-19 was higher than that of many other healthcare providers (Kishk et al., 2021). A systematic review of factors influencing well-being among healthcare assistants, such as NAs, concluded that most of the studies investigated individual factors rather than organizational factors (Norful et al., 2024). Even among studies focusing on organizational factors, researchers tended to examine workplace characteristics like workload and shift hours, rather than perceived workplace environment and outcomes such as burnout and general distress. A review examining perceived workplace environments found 24 studies exploring this relationship with nursing organizational outcomes (e.g., satisfaction, intent to leave, burnout, and work engagement), but none included specific mental health outcomes such as depression, anxiety, and somatic symptoms (Swiger et al., 2017). The study explores perceived workplace environments and specific psychological distress symptoms, not just general distress.

Guided by Lazarus and Folkman’s (1984) Stress and Coping Theory, this study examines the association between perceived workplace environment and psychological distress (stress, depressive, anxiety, and somatic symptoms) in inpatient RNs and NAs during the COVID-19 pandemic. This theoretical approach underscores the importance of addressing modifiable environmental factors to enhance the overall resilience and well-being of the nursing workforce. Exploring the relationship between the workplace environment and the psychological distress of nursing staff can inform interventions designed to improve mental health and retention among nursing staff.

## Methods

The study, part of a larger multimethod project (Killela et al., 2025), used a descriptive cross-sectional design with a quantitative survey to describe RNs’ and NAs’ perceived workplace environment and psychological distress symptoms during the COVID-19 pandemic.

### Setting and Participants

Data were collected from a large academic medical center in the southeastern United States, which has over 900 inpatient beds. The study received approval from the University of North Carolina at Chapel Hill Institutional Review Board (IRB #20-2323) and the hospital nursing research council. Inclusion criteria included full-time RNs or NAs employed at the medical center since at least February 2020 and who spent >80% of their clinical time delivering direct patient bedside care.

### Data Collection

A convenience sample was recruited through informational flyers, hospital listservs, and in-person staff meetings on both COVID-19 intensive units and units that did not focus on COVID-19 patients. Data were collected via an online Qualtrics survey between April 2021 and September 2021. The survey was piloted for issues and clarity by an RN not employed at the medical center. After completing eligibility screening and an embedded consent process, participants completed questionnaires on demographics, perceived workplace environment, and psychological distress symptoms. The informed consent process assured participants that their responses would remain anonymous and that survey data would only be shared in aggregate form. To protect anonymity, the survey was conducted on a secure, University-hosted platform, and all data were stored on confidential, secure servers. Email addresses were collected voluntarily only from participants interested in entering a raffle for one of ten $30 gift cards or participating in follow-up interviews. These email addresses were stored separately from survey responses and deleted after the study was completed. IP addresses were not collected, and all efforts were made to ensure participant privacy and confidentiality throughout the study.

### Measures

Individual characteristics included age, gender identity, race, ethnicity, education, work unit, years of nursing experience, years of unit experience, and clinical ladder role. Workplace environment was measured using the Practice Environment Scale of the Nursing Work Index (PES-NWI; Lake, 2002), including a composite score and five subscales: Nurse Participation in Hospital Affairs (Participation), Nursing Foundations for Quality of Care (Care), Nurse Manager Ability, Leadership and Support (Leadership), Staffing and Resource Adequacy (Staffing), and Collegial Nurse–Physician Relations (Collegiality). Psychological distress included perceived stress (PSS-10; Cohen et al., 1983), anxiety (Generalized Anxiety Disorder Questionnaire [GAD-7]; Spitzer et al., 2006), depression (Patient Health Questionnaire-9 [PHQ-9]; Kroenke et al., 2001), and somatic symptoms (PHQ-15; Kroenke et al., 2002). For our sample reliability estimates for all scales ranged from acceptable to excellent (Cronbach’s α > .70), except for the PES-NWI Staffing subscale, which demonstrated poor reliability (Cronbach’s α = .51). Detailed information on the measures, scoring, and internal reliability of workplace predictors and psychological distress outcomes for this sample is provided in Table 1.

**Table 1.** Summary of Study Measures, Subscales, Scoring, and Reliability Estimates.

Domain or concept	Measure	Items, response scale, and notes	Scoring	Sample internal reliability (α)
Demographics	Self-report	Age, gender identity, race, ethnicity	—	—
Nursing characteristics	Clinical ladder roles	Entry-level NA I, NA II, CN I (new graduate nurse), CN II, CN III, CN IV (assistant manager)	—	—
	Highest level of nursing education	NA I, NA II, Diploma in Nursing, ADN, BSN, MSN	—	—
Workplace predictors				
Workplace environment	PES-NWI (Lake, 2002)	31 items, 1–4 Likert Extent to which an individual agrees that the item is present in their current job (1 = strongly agree to 4 = strongly disagree) Modified for NA participants by replacing the term “nurse” with “nursing assistant” in relevant items. The PES-NWI is one of the most widely used tools to measure nursing practice environments, with multiple studies providing evidence of reliability and validity (Swiger et al., 2017).	Composite = average of subscales; ≥2.5 = favorable	0.90
Facility-level nurse participation in hospital affairs	PES-NWI participation subscale	9 items, 1–4 Likert	Subscale mean	0.83
Facility-level nursing foundations for quality of care	PES-NWI care subscale	10 items, 1–4 Likert	Subscale mean	0.75
Unit-level nurse manager ability, leadership and support	PES-NWI leadership subscale	5 items, 1–4 Likert	Subscale mean	0.74
Staffing and resource adequacy	PES-NWI staffing subscale	4 items, 1–4 Likert	Subscale mean	0.51
Collegial nurse–physician relations	PES-NWI collegiality subscale	3 items, 1–4 Likert	Subscale mean	0.84
Psychological distress outcomes				
Perceived stress	The PSS (Cohen et al., 1983)	10 items, 1–5 Likert Frequency of specific stress-related thoughts and feelings in the past month (1 = never to 5 = almost always). The PSS is a widely used measure of perceived stress with acceptable psychometric properties and has been successfully applied to previous samples of nurses (Abdollahi et al., 2014).	Sum (10–50); higher = more stress	0.85
Anxiety symptoms	GAD-7 (Spitzer et al., 2006)	7 items, 0–3 Likert Frequency in which they experienced the anxiety symptoms over the past 2 weeks (0 = not at all to 3 = nearly every day).	Sum (0–21) Severity: minimal (0–4), mild (5–9), moderate (10–14), severe (15–21)	0.92
Depressive symptoms	PHQ-9 (Kroenke, et al., 2001)	9 items, 0–3 Likert Frequency in which they experienced the depressive symptom over the past 2 weeks (0 = not at all to 3 = nearly every day). The PHQ-9 has been utilized in previous studies to assess depressive symptoms among nursing staff delivering care to COVID-19 patients (Kang et al., 2020).	Sum (0–27) Severity: minimal (0–4), mild (5–9), moderate (10–14), moderately severe (15–19), severe (20–27)	0.82
Somatic symptoms	PHQ-15 (Kroenke et al., 2002).	15 items, 0–2 Likert How much individuals were troubled by specific symptoms over the past 4 weeks (0 = not bothered at all to 2 = bothered a lot). Somatic symptoms involving the cardiopulmonary and gastrointestinal systems, as well as generalized pain and fatigue.	Sum (0–30) Severity: mild (0–4), low (5–9), medium (10–14), high (15–30)	0.77

*Note.* ADN = Associate Degree in Nursing; BSN = Bachelor of Science in Nursing; MSN = Master of Science in Nursing; NA = Nursing Assistant; CN = Clinical Nurse; PES-NWI = Practice Environment Scale Nursing Work Index; PSS = Perceived Stress Scale; GAD-7 = Generalized Anxiety Disorder Questionnaire; PHQ = Patient Health Questionnaire.

### Analysis

Descriptive statistics summarized demographics, perceived workplace environment, and psychological distress symptoms. Independent t-tests compared RNs and NAs workplace environment and symptom scores. A stepwise approach was employed to assess the relationship between workplace environment and psychological distress. Univariate linear regression models were run for each workplace environment score (total score and 5 subscales) and key demographic variables (age, gender, race, clinical ladder, and COVID-19 unit). For each of the workplace environment scores that were significant (α = .05) in the univariate models, an adjusted model was subsequently conducted, incorporating any significant demographic variables from the univariate analysis. This stepwise approach limited the number of predictor variables in the models given the sample size (*n* = 84).

## Results

### Sample Demographics

The cohort comprised 84 participants (14 NAs, 70 RNs) with a mean age of 35.8 years (Standard deviation [*SD*] = 10.2). All participants identified as cisgender, with the majority as female (*n* = 77) and 7 as male. Sixty participants identified as White, 17 as Black/African American, 5 as Asian American, 1 as Native American, and 1 as Mixed Race. Four participants identified as Hispanic/Latine/Latinx.

Among RNs, 5 had a master’s degree, 44 a bachelor’s degree, 19 an associate degree, and 2 a diploma. Participants averaged 10.34 years of overall experience (*SD* = 8.79) and 5.86 years of unit-specific experience (*SD* = 5.94). Eleven participants (13.1%) worked on COVID-19 designated units, while 73 (86.9%) worked on non-COVID units. Regardless of unit designation, 36.9% of participants reported providing more than 10% of their care to COVID-positive patients, indicating that COVID-19 care spanned the hospital.

### Perceived Workplace Environment

The sample average PES-NWI scores were 2.9 for RNs (*SD* = 0.35) and 2.87 for NAs (*SD* = 0.32). The PES-NWI subscale mean scores were highest for Care (3.1, *SD* = 0.3) and Leadership (3.1, *SD* = 0.5), followed by Collegiality (2.9, *SD* = 0.6), Participation (2.8, *SD* = 0.5), and lowest, yet positive, Staffing (2.5, *SD* = 0.5).

### Psychological Distress Symptoms

The sample average stress (PSS) scores were 22.2 for RNs (*SD* = 2.9) and 22.5 for NAs (*SD* = 2.06). The sample average depressive (PHQ-9) scores were 7.24 for RNs (*SD* = 4.6) and 6.29 for NAs (*SD* = 3.83), indicating mild severity. The sample average anxiety (GAD-7) scores were 7.8 for RNs (*SD* = 5.3) and 5.86 for NAs (*SD* = 4.5), indicating mild severity. The sample average somatic symptoms (PHQ-15) scores were 8.36 for RNs (*SD* = 4.19) and 11 for NAs (*SD* = 4.77), indicating mild severity for RNs and moderate for NAs. The average scores for symptoms across the outcomes indicated mild or moderate severity and did not meet clinical cutoff data. However, 33.3% reported moderate or severe anxiety symptoms, 28.5% reported moderate or moderate–severe depressive symptoms, and 42.1% reported moderate or severe somatic symptoms (see Table 2).

**Table 2.** Cohort Demographics, Workplace Environment, and Psychological Distress Symptoms.

	Total sample		Nurses		Nursing assistants	
Variable	*n*	Mean (*SD*) or %	*n*	Mean (*SD*) or %	*n*	Mean (*SD*) or %
Age	83*	35.8 (10.2)	69	36.4 (10.3)	14	32.9 (9.7)
Gender	84		70		14	
Female	77	91.7	63	90	14	100
Male	7	8.3	7	10	0	
Race	84		70		14	
White	60	71.4	54	77.1	6	42.8
Black‎/African American	17	20.2	10	14.3	7	50
Asian American	5	6	5	7.1	0	
Native American	1	1.2	1	1.4	0	
Mixed Race (White/Black‎/African American‎/Native American)	1	1.2	0		1	7.2
Ethnicity	84		70		14	
Hispanic or Latinx	4	4.8	4	5.7	0	
NOT Hispanic or Latinx	80	95.2	66	94.3	14	100
Role	84		70		14	
Nurse	70	16.7	70	100	—	—
Nursing assistant	14	83.3	—	—	14	100
Clinical ladder	84		70		14	
NA I	9	10.7	—	—	9	64.3
NA II	6	7.1	—	—	5	35.7
CN I	3	3.6	4	6	—	—
CN II	46	54.8	46	66	—	—
CN III	15	17.9	15	21	—	—
CN IV	5	6	5	7	—	—
Education	84		70		14	
NA I	9	10.7	—	—	9	64.3
NA II	5	6	—	—	5	35.7
Diploma in nursing	2	2.4	2	2.9	—	—
and	19	22.6	19	27.1	—	—
BSN	44	52.4	44	62.9	—	—
MSN	5	6	5	7.1	—	—
Certification	70		70		—	—
Yes	38	54.3	38	54.3	—	—
No	32	45.7	32	45.7	—	—
Years of experience*	75	10.3 (8.8)	61	10.3 (9.0)	14	10.5 (7.9)
Years of unit experience*	70	5.9 (5.9)	59	6.2 (6.3)	11	4.2 (2.8)
Unit type	84		70		14	
Medical surgical	28	33.3	21	30	7	50
Step down	5	6	5	7.1	0	
Intensive care unit	19	22.6	17	24.3	2	14.3
Women’s	10	11.9	9	12.9	1	7.1
Pediatrics	15	17.9	12	17.1	3	21.4
Psychiatry	6	7.1	5	7.1	1	7.1
Emergency	1	1.2	1	1.4	—	—
COVID unit	84					
Yes	11	13.1	9	87.1	1	7.1
No	73	86.9	61	12.9	13	92.9
COVID care						
<10% patients	53	63.1	51	72.9	2	16.7
>10% patients	31	36.9	19	27.1	12	83.3
PES-NWI total score	84	2.9 (0.3)	70	2.9 (0.3)	14	2.9 (0.3)
PES-NWI nurse participation in hospital affairs	84	2.8 (0.5)	70	2.8 (0.5)	14	2.9 (0.4)
PES-NWI nursing foundations for quality of care	84	3.1 (0.3)	70	3.0 (0.3)	14	3.1 (0.3)
PES-NWI nurse manager ability, leadership, and support for nurses	84	3.1 (0.5)	70	3.1 (0.5)	14	3.1 (0.5)
PES-NWI staffing and resource adequacy	84	2.5 (0.5)	70	2.5 (0.5)	14	2.5 (0.4)
PES-NWI collegial nurse–physician relations		2.9 (0.6)	70	3.0 (0.5)	14	2.3 (0.7)
PSS	84	22.3 (2.8)	70	22.2 (2.9)	14	22.5 (2.1)
GAD-7	84	7.5 (5.2)	70	7.8 (5.3)	14	5.9 (4.5)
None (0–4)	26	31.0	20	28.6	6	42.9
Mild (5–9)	30	35.7	26	37.1	4	28.6
Moderate (10–14)	19	22.6	15	21.4	4	28.6
Severe (15–21)	9	10.7	9	12.9	0	
PHQ-9	84	7.1 (4.5)	70	7.2 (4.6)	14	6.3 (3.8)
None (0–4)	28	33.3	24	34.3	4	28.6
Mild (5–9)	32	38.1	25	35.7	7	50
Moderate (10–14)	18	21.4	15	21.4	3	21.4
Moderate severe (15–19)	6	7.1	6	8.6	0	
PHQ-15	84	8.8 (4.4)	70	8.4 (4.2)	14	11 (4.8)
None (0–4)	12	14.3	12	17.1	0	
Mild (5–9)	36	43.4	29	41.4	7	50
Moderate (10–14)	31	36.1	26	37.1	5	35.7
Severe (15–30)	5	6	3.0	4.3	2	14.3

*Note.* NA = Nursing Assistant; CN = Clinical Nurse; CNA = Certified Nursing Assistant; ADN = Associate Degree in Nursing; BSN = Bachelor of Science of Nursing; MSN = Master of Science in Nursing; Certification = American Nurses Credentialing Center (ANCC) Board Certified RN; PES-NWI = Practice Environment Scale Nursing Work Index; PSS = Perceived Stress Scale; GAD-7 = Generalized Anxiety-7; PHQ-9 = Patient Health Questionnaire-9; PHQ-15 = Patient Health Questionnaire-15; SD = Standard deviation.

* missing demographic data: age *n* = 1, years of experience *n* = 9, years of unit experience *n* = 14.

### Independent *t*-Tests: RN and NA Differences

Independent t-tests found NAs rated their PES-NWI Collegiality subscale experience 0.74 points lower than RNs (*t* = −3.84, 95% confidence interval [−1.15, −0.33], *p* = .001). No other significant differences were found in the PES-NWI total score, its other subscales, or in any measures of psychological distress symptoms between the two groups (see Supplemental Table 1).

### Linear Regression: Perceived Stress

Univariate models showed that a one-point increase in the PES-NWI Total, Staffing subscale, and Participation subscale scores was associated with lower perceived stress scores by 3.92 points (*p* = .039), 3.82 points (*p* = .006), and 2.96 points (*p* = .006), respectively. The other subscales (Care, Leadership, Collegiality) were not significantly associated with perceived stress. Among the demographic characteristics, identifying as non-white was associated with lower stress score than white participants. In the three adjusted models, the PES-NWI Total, Staffing, and Participation subscale scores were each no longer significantly associated with perceived stress once race was added to the models (Table 3).

**Table 3.** Univariate and Adjusted Linear Regression Results for Stress Symptoms (Perceived Stress Scale).

	Univariate			Adjusted model 1 (total score)			Adjusted model 2 (staffing)			Adjusted model 3 (participation)		
Variable	Estimate	95% *CI*	*p*-value	Estimate	95% *CI*	*p*-value	Estimate	95% *CI*	*p*-value	Estimate	95% *CI*	*p*-value
PES-NWI total score	−**3.92**	**[**−**7.63**, −**0.21]**	.**0 39**	−1.42	[−5.34, 2.5]	.473						
PES-NWI care	−2.32	[−6.17, 1.53]	.233									
PES-NWI leadership	−1.63	[−4.35, 1.08]	.234									
PES-NWI staffing	−**3.82**	**[**−**6.53**, −**1.11]**	.**006**				−2.61	[−5.29, 0.06]	.056			
PES-NWI collegiality	−0.57	[−2.63, 1.48]	.58									
PES-NWI participation	−**2.96**	**[**−**5.73**, −**0.19]**	.**036**							−1.55	[−4.35, 1.25]	.273
Age	−**0.17**	**[**−**0.29**, −**0.04]**	.**008**	−0.11	[−0.24, 0.02]	.099	−0.11	[−0.23, 0.01]	.065	−0.11	[−0.23, 0.02]	.103
Gender (ref = male)												
Female	2.95	[−1.67, 7.57]	.207									
Race (ref = white)												
Non-White	−**4.47**	**[**−**7.15**, −**1.79]**	.**001**	−**3.37**	**[**−**6.16**, −**0.58]**	.**019**	−**2.92**	**[**−**5.69**, −**0.15]**	.**039**	−**3.35**	**[**−**6.12**, −**0.59]**	.**018**
Clinical ladder (ref = CN I/II)												
CNA I or II	−1.56	[−5.05, 1.93]	.377									
CN III or IV	0.28	[−2.86, 3.42]	.859									
COVID floor (ref = non COVID floor)												
COVID floor	0.9	[−3.08, 4.88]	.654									

*Note.* PES-NWI = The Practice Environment Scale Nursing Work Index; CN = Clinical Nurse; CNA = Clinical Nursing Assistant; CI = Confidence interval.

Bold = significant alpha < .05.

### Linear Regression: Anxiety Symptoms

Univariate models showed that a one-point increase in PES-NWI Total and Participation subscale scores was associated with lower anxiety scores by 3.42 points (*p* = .041) and 2.96 points (*p* = .009), respectively. Among the demographic characteristics, older age and non-white race were associated with lower anxiety scores, while female gender was associated with higher anxiety scores. In the two adjusted models, PES-NWI Total and Participation subscale scores were no longer significantly associated with anxiety symptoms once age, gender, and race were added to the models (Table 4).

**Table 4.** Univariate and Adjusted Linear Regression Results for Anxiety Symptoms (Generalized Anxiety Scale-7).

	Univariate			Adjusted model 1 (total score)			Adjusted model 2 (participation)		
Variable	Estimate	95% *CI*	*p*-value	Estimate	95% *CI*	*p*-value	Estimate	95% *CI*	*p*-value
PES-NWI total score	−**3.42**	**[**−**6.69**, −**0.14]**	.**041**	−1.31	[−4.7, 2.08]	.445			
PES-NWI care	−2.97	[−6.33, 0.39]	.083						
PES-NWI leadership	−2.17	[−4.53, 0.2]	.072						
PES-NWI staffing	−1.79	[−4.25, 0.68]	.154						
PES-NWI collegiality	0.18	[−1.63, 2]	.84						
PES-NWI participation	−**2.96**	**[**−**5.38**, −**0.54]**	.**01**				−1.67	[−4.06, 0.73]]	.171
Age	−**0.19**	**[**−**0.29**, −**0.08]**	.**001**	−**0.13**	**[**−**0.24**, −**0.01]**	.**027**	−**0.12**	**[**−**0.23**, −**0.01]**	.**03**
Gender (ref = male)									
Female	**4.26**	**[0.25, 8.26]**	.**037**	**4.34**	**[0.56, 8.11]**	.**025**	**4.29**	**[0.57, 8.01]**	.**024**
Race (ref = white)									
Non-White	−**3.23**	**[**−**5.65**, −**0.82]]**	.**009**	−**2.39**	**[**−**4.8, 0.01]**	.**05**	−**2.35**	**[**−**4.73, 0.03]**	.**052**
Clinical ladder (ref = CN I/II)									
CNA I or II	−1.3	[−4.38, 1.77]	.401						
CN III or IV	0.58	[−2.18, 3.34]	.678						
COVID floor (ref = non COVID floor)									
COVID floor	1.28	[−2.22, 4.77]	.47						

*Note.* PES-NWI = The Practice Environment Scale Nursing Work Index; CN = Clinical Nurse; CNA = Clinical Nursing Assistant; CI = Confidence interval.

Bold = significant alpha < .05.

### Linear Regression: Depressive Symptoms

Univariate models showed that a one-point increase in the PES-NWI Total, Care subscale, Leadership subscale, Staffing subscale, and Participation subscale scores was associated with lower depressive symptom scores by 4.15 points (*p* = .028), 3.68 points (*p* = .015), 3.21 points (*p* = .022), 2.89 points (*p* = .010), and 2.54 points (*p* = .007), respectively. The PES-NWI Collegiality subscale was not significantly associated with depressive symptom score. Among the demographic characteristics, older age was associated with lower (better) depressive symptom scores, while female gender was associated with higher (worse) depressive symptom scores. In the five adjusted models, the Leadership subscale score was the only workplace environment measure that remained significantly associated with depressive symptoms after controlling for age and gender. As Leadership scores improved, depressive scores also improved (lowered). For complete adjusted model results, see Table 5.

**Table 5.** Univariate and Adjusted Linear Regression Results for Depressive Symptoms (Patient Health Questionnaire-9).

	Univariate			Adjusted model 1 (total score)			Adjusted model 2 (care)			Adjusted model 2 (leadership)			Adjusted model 4 (staffing)			Adjusted model 5 (participation)		
Variable	Estimate	95% *CI*	*p*-value	Estimate	95% *CI*	*p-*value	Estimate	95% *CI*	*p-*value	Estimate	95% *CI*	*p-*value	Estimate	95% *CI*	*p-*value	Estimate	95% *CI*	*p-*value
PES-NWI total score	−**4.04**	**[**−**6.78**, −**1.29]**	.**004**	−2.81	[−5.68, 0.06]	.055												
PES-NWI care	−**3.39**	**[**−**6.24**, −**0.55]**	.**02**				−1.87	[−4.87, 1.13]	.219									
PES-NWI leadership	−**2.58**	**[**−**4.57**, −**0.58]**	.**012**							−**2.03**	**[**−**4.01**, −**0.04]**	.**045**						
PES-NWI staffing	−**2.33**	**[**−**4.41**, −**0.24]**	.**029**										−1.68	[−3.64, 0.28]	.093			
PES-NWI collegiality	−0.44	[−1.99, 1.12]	.576															
PES-NWI participation	−**2.92**	**[**−**4.97**, −**0.86]**	.**006**													−1.98	[−4.04, 0.08]	.059
Age	−**0.17**	**[**−**0.26**, −**0.08]**	**0**	−**0.12**	**[**−**0.22**, −**0.02]**	.**016**	−**0.13**	**[**−**0.23**, −**0.03]**	.**01**	−**0.13**	**[**−**0.22**, −**0.04]**	.**007**	−**0.15**	**[**−**0.23**, −**0.06]**	.**002**	−**0.13**	**[**−**0.22**, −**0.04]**	.**007**
Gender (ref = male)																		
Female	**3.99**	**[0.56, 7.41]**	.**023**	**3.85**	**[0.63, 7.06]**	.**02**	**3.69**	**[0.44, 6.95]**	.**02**	**4**	**[0.77, 7.23]**	.**016**	**3.39**	**[0.19, 6.59]**	.**038**	**3.6**	**[0.42, 6.79]**	.**027**
Race (ref = white)																		
Non-White	−1.98	[−4.1, 0.13]	.066															
Clinical ladder (ref = CN I/II)																		
CNA I or II	−1.2	[−3.84, 1.44]	.369															
CN III or IV	−0.98	[−3.35, 1.39]	.413															
COVID floor (ref = non COVID floor)																		
COVID floor	−0.32	−3.34 2.69	0.832															

*Note.* PES-NWI = The Practice Environment Scale Nursing Work Index; CN = Clinical Nurse; CNA = Clinical Nursing Assistant; CI = Confidence interval.

Bold = significant alpha < .05.

### Linear Regression: Somatic Symptoms

In the univariate models, a one-point increase in the PES-NWI Total, Leadership subscale, and Participation subscale scores was associated with lower somatic symptom scores by 3.45 points (*p* = .025), 2.87 points (*p* = .018), and 2.63 points (*p* = .012), respectively. The Care, Staffing, and Collegiality subscale scores were not significantly associated with somatic symptom scores. Among the demographic characteristics, older age was associated with lower (better) somatic symptom scores, while identifying as a female and being an NA (compared to being a CNI/II or CNIII/IV) were associated with higher (worse) somatic symptom scores. In the three adjusted models, the Leadership subscale score was the only workplace environment measure that remained significantly associated with somatic symptoms after controlling for age, gender, and clinical ladder. As PES-NWI Leadership scores improved, somatic symptom scores also improved (lowered). For complete adjusted model results, see Table 6.

**Table 6.** Univariate and Adjusted Linear Regression Results for Somatic Symptoms (Patient Health Questionnaire-15).

	Univariate			Adjusted model 1 (total score)			Adjusted model 2 (leadership)			Adjusted model 3 (participation)		
Variable	Estimate	95% *CI*	*p*-value	Estimate	95% *CI*	*p*-value	Estimate	95% *CI*	*p*-value	Estimate	95% *CI*	*p*-value
PES-NWI total score	−**2.78**	**[**−**5.54**, −**0.02]**	.**048**	−2.22	[−5.17, 0.73]	.138						
PES-NWI care	−0.91	[−3.78, 1.96]	.529									
PES-NWI leadership	−**2.1**	**[**−**4.08**, −**0.13]**	.**037**				−**2.11**	**[**−**4.16**, −**0.06]**	.**044**			
PES-NWI staffing	−1.34	[−3.42, 0.74]	.204									
PES-NWI collegiality	−1.27	[−2.77, 0.23]	.095									
PES-NWI participation	−**2.2**	**[**−**4.25**, −**0.15]**	.**036**							−1.78	[−3.88, 0.32]	.095
Age	−**0.12**	**[**−**0.21**, −**0.02]**	.**014**	−0.06	[−0.16, 0.04]	.234	−0.06	[−0.16, 0.03]	.197	−0.06	[−0.16, 0.03]	.201
Gender (ref = male)												
Female	**4.61**	**[1.3, 7.91]**	.**007**	**4.19**	**[0.84, 7.55]**	.**015**	**4.35**	**[1.03, 7.68]**	.**011**	**4.01**	**[0.68, 7.34]**	.**019**
Race (ref = white)												
Non-White	0.11	[−2, 2.22]	.918									
Clinical Ladder (ref = CN I/II)												
CNA I or II	**2.75**	**[0.23, 5.26]**	.**033**	2.08	[−0.37, 4.53]	.095	2.21	[−0.22, 4.64]	.074	2.19	[−0.26, 4.64]	.079
CN III or IV	−0.34	[−2.6, 1.93]	.769	−0.01	[−2.35, 2.32]	.992	0.27	[−2.08, 2.61]	.82	−0.04	[−2.35, 2.28]	.975
COVID floor (ref = non COVID floor)												
COVID floor	0.57	[−2.38, 3.52]	.701									

*Note.* PES-NWI = The Practice Environment Scale Nursing Work Index; CN = Clinical Nurse CNA = Clinical Nursing Assistant; CI = Confidence interval.

Bold = significant alpha < .05.

## Discussion

This study explored how RNs and NAs perceived their workplace environment and psychological distress symptoms (stress, depression, anxiety, and somatic) during COVID-19, providing insights that extend beyond the pandemic. COVID-19 served as a real-world example of a high-stress environment, highlighting significant links between better workplace environment scores and lower psychological distress symptoms. Many associations lost significance after demographic adjustments, likely due to small sample size and limited power, especially with only 14 nursing assistants. Larger studies are needed to confirm these findings and better understand how workplace conditions impact psychological distress. Future crises, such as climate-driven natural disasters and global political conflicts, are likely to increase workplace stress for nurses. These findings can guide strategies to build resilience and improve workplace environments during high-stress conditions.

Notable findings include differences in nurse–physician relationships, with NAs reporting significantly worse relations with physicians compared to RNs. Previous research has linked poor nurse–physician relationships to negative outcomes, such as higher nurse turnover and increased missed nursing care (Stemmer et al., 2022). Limited direct interaction may explain these differences, as NAs often communicate with physicians through RNs. Further research is needed to assess the impact of physician–NA relationships on NAs’ well-being, organizational dynamics, and patient outcomes.

RNs and NAs showed no significant differences in psychological distress, though about one-third reported moderate to severe symptoms, indicating a need for further assessment and intervention. NAs had slightly more somatic symptoms, with a *p-*value close to significance (0.07). This may be linked to their physically demanding roles, such as lifting and repositioning patients, with surveys of NAs in acute care settings identifying physical strain (64.6%) as a major challenge (Miller et al., 2024). Additionally, NAs faced greater exposure and infection risk during COVID-19 (Kishk et al., 2021), which could contribute to somatic symptoms, workplace injury, and staff turnover (McCaughey et al., 2014; Miller et al., 2024). Addressing psychological distress among nursing staff is a critical clinical priority, as neglecting it raises the risk of mental health problems, potentially jeopardizing their well-being and increasing workforce attrition.

These findings highlight the workplace environment’s role in psychological distress for RNs and NAs, with NAs experiencing similar or slightly higher distress levels. While the impact of poor work environments on RNs is well-documented, NAs are often overlooked despite facing similar stressors. Interventions aimed at enhancing RNs’ well-being should also consider NAs, by involving them in the planning and execution of these programs, and addressing their unique individual and organizational needs (Norful et al., 2024).

Unadjusted models showed that higher PES-NWI total scores were associated with lower psychological distress symptoms, highlighting the need to improve unit- and facility-level workplace environments. Adjusted models identified nurse manager leadership as a key predictor of reduced depressive and somatic symptoms. A systematic review suggests that relational-focused nurse leadership styles (transformational, authentic, ethical, and servant) were positively associated with nurses’ work-related well-being (Niinihuhta & Häggman-Laitila, 2022). Nurse managers are vital to achieving organizational goals, but increasingly complex acute care settings have expanded their responsibilities, creating challenges in managing their duties effectively (Schlotzhauer et al., 2023). Nurse managers reported many challenges during COVID-19, such as heavy workloads, ethical dilemmas, poor crisis management, and high stress and burnout (Chipps et al., 2022; Ozmen & Arslan Yurumezoglu, 2022). Evidence-based interventions have been identified to improve nurse manager competence and job satisfaction (Niskala et al., 2020). Supporting nurse managers benefits staff and organizations by improving care quality, satisfaction, stress management, and turnover rates (Alanazi et al., 2023; Niinihuhta & Häggman-Laitila, 2022; Warshawsky et al., 2022). However, individual-level interventions alone may be insufficient. Structural and organizational support is essential to help nurse managers address workplace challenges and effectively manage their units (Warshawsky et al., 2022).

Regression analyses revealed associations between demographic factors (age, gender, and race) and psychological distress symptoms. Older nurses reported lower psychological distress symptoms, consistent with research suggesting age is a protective factor against intent to leave (de Vries et al., 2023). The average participant age (35.6 years) was lower than the national median (46 years), which may reflect sampling bias, as younger nurses may be more comfortable with online surveys, or the significant loss of over 200,000 experienced RNs during the pandemic (Smiley et al., 2023). Female participants reported higher anxiety, depression, and somatic symptoms, aligning with findings that female nurses report lower levels of resilience compared to male nurses (Huang et al., 2020). Non-white participants in our study reported lower stress and anxiety compared to white participants, despite prior research suggesting racially minoritized nurses experienced higher COVID-19-related mortality and workplace racism (Boserup et al., 2020; National Nurses United, 2020; Robert Wood Johnson Foundation, 2023). While some studies report modest racial/ethnic differences in well-being, findings are inconsistent, and gaps remain in understanding race-specific stressors and coping strategies among minority nurses (Abrahim & Holman, 2023). Other structural and individual factors, such as mental health care access, social support, or coping mechanisms, may contribute to distress disparities (Al-Amin et al., 2024). Although 20% of the sample identified as Black or African American, the small sample may still introduce sampling bias and limit generalizability. These findings could reflect unique coping strategies or protective factors among minoritized nurses, warranting further investigation.

This study has several limitations to consider. It relied on a convenience sample that included only a small proportion of the target hospital nursing staff, especially few NAs, limiting statistical power and generalizability. Low participation rates may reflect concerns about confidentiality or fear of retribution for reporting personal or workplace challenges, potentially underestimating workplace concerns and psychological distress. While the survey was conducted on a secure platform with voluntary email collection and assurances of anonymity, some participants may have been hesitant to participate, especially those experiencing higher levels of distress. Additionally, omissions about work or unit-specific experiences may reflect efforts to avoid sharing identifiable information. Despite these limitations, the inclusion of NAs, an often-underrepresented group in workplace well-being research, provides valuable insights.

Data collection occurred from April to September 2021 during a specific phase of the pandemic. Since then, COVID-19 has become endemic. The 6-month data collection period likely captured fluctuating stressors related to COVID-19, as the virus’s impacts evolved throughout the pandemic. Participants reflected on their experiences retrospectively, and responses may have varied depending on specific phases, such as before vaccine rollout or personal protective equipment (PPE) availability. While this study provides valuable insights into nursing well-being during a critical period, its small sample size, specific timeframe, and the evolving nature of COVID-19 are limitations to consider.

Our findings underscore several key areas for future research to advance understanding of workplace stressors, psychological distress, and the well-being of nursing staff during crisis. Future studies should recruit larger, diverse samples of RNs and NAs to improve representation, statistical power, and generalizability. Longitudinal studies are needed to assess the long-term psychological effects of crises on healthcare workers, including coping, post-traumatic stress, burnout, and resilience. Research should also examine organizational outcomes, such as patient safety, care quality, intent to leave, and workforce attrition, alongside evidence-based interventions to reduce workplace stressors, enhance manager support, and improve crisis preparedness. Studies should examine how nurse managers mitigate nursing staff distress and influence both staff and patient outcomes. Expanding research beyond this study’s focus on one crisis is key to developing effective strategies to support the nursing workforce across crises. Future research can offer insights to shape policies and strategies that strengthen nursing staff resilience and well-being, improving healthcare outcomes in crises.

## Conclusion

This study highlights how RNs and NAs perceive their workplace environment and its relationship to psychological distress. Both groups reported similar levels of distress, and unadjusted analyses showed that workplace environment was linked to distress outcomes. Adjusted models identified the Nurse Manager’s Ability, Leadership, and Support subscale as significantly associated with lower depressive and somatic symptoms. These findings emphasize the need to improve both unit- and hospital-level environments, with nurse managers playing a central role. This research can guide interventions to reduce distress, safeguard well-being, strengthen the nursing workforce, and enhance organizational outcomes during future crises.

## Supplemental Material

sj-docx-1-cnr-10.1177_10547738261429339 – Supplemental material for Workplace Environment and Psychological Distress of Nurses and Nursing Assistants During COVID-19 Supplemental material, sj-docx-1-cnr-10.1177_10547738261429339 for Workplace Environment and Psychological Distress of Nurses and Nursing Assistants During COVID-19 by Harry Adynski, Cassandra Dictus, Mary K. Killela, Gillian I. Adynski, Elizabeth Allen Myer, Leah Morgan, Hayden Hmiel and Jessica R. Williams in Clinical Nursing Research
