# Supplementary material for: Workplace Environment and Psychological Distress of Nurses and Nursing Assistants During COVID-19
Source: Clin Nurs Res. 2026 Mar 23;35(4):200–14. doi: 10.1177/10547738261429339 (PMC13150001; doi:10.1177/10547738261429339)
Supplement: sj-docx-1-cnr-10.1177_10547738261429339 – Supplemental material for Workplace Environment and Psychological Distress of Nurses and Nursing Assistants During COVID-19 [file sj-docx-1-cnr-10.1177_10547738261429339.docx]

**Table Description:** This table presents the results of independent t-tests, comparing differences in workplace environment and psychological distress measures between nurses and nursing assistants within the sample.

**Supplemental Table 1:** *Nurse and Nursing Assistant t-test Results*

| Variable | **Mean Difference** | **t-statistic** | **95% CI** | **95% CI** | **p-value** |
| --- | --- | --- | --- | --- | --- |
| PES-NWI Total Score | -0.03 | -0.32 | -0.23 | 0.17 | 0.755 |
| PES-NWI Nurse Participation in Hospital Affairs | 0.06 | 0.47 | -0.21 | 0.34 | 0.643 |
| PES-NWI Nursing Foundations for Quality of Care | 0.05 | 0.59 | -0.13 | 0.23 | 0.563 |
| PES-NWI Nurse Manager Ability, Leadership, and Support for Nurses | 0.00 | -0.02 | -0.32 | 0.32 | 0.985 |
| PES-NWI Staffing and Resource Adequacy | 0.02 | 0.16 | -0.25 | 0.3 | 0.872 |
| PES-NWI Collegial Nurse-Physician Relations | **-0.74** | **-3.84** | **-1.15** | **-0.33** | **0.001** |
| PSS | -1.41 | -0.88 | -4.75 | 1.92 | 0.388 |
| PHQ-9 | -0.96 | -0.82 | -3.37 | 1.46 | 0.419 |
| GAD-7 | -1.94 | -1.43 | -4.77 | 0.89 | 0.168 |
| PHQ-15 | 2.64 | 1.93 | -0.24 | 5.53 | 0.07 |
| Note: PES-NWI = The Practice Environment Scale Nursing Work Index, PSS = Perceived Stress Scale, GAD-7= Generalized Anxiety-7, PHQ-9 = Patient Health Questionnaire - 9, PHQ-15= Patient Health Questionnaire –15, CI= Confidence Interval. **Bold = significant alpha<0.05.** | | | | | |
